# Dietary Protein Intake and Type 2 Diabetes Among Women and Men in Northeast China

**DOI:** 10.1038/srep37604

**Published:** 2016-11-29

**Authors:** Jie Li, Changhao Sun, Simin Liu, Ying Li

**Affiliations:** 1 National Key Discipline, Department of Nutrition and Food Hygiene, School of Public Health, Harbin Medical University, Harbin, China; 2 Departments of Epidemiology and Medicine, Center for Global Cardiometabolic Health, Brown University, Providence, RI USA

## Abstract

We conducted a comprehensive and in-depth assessment of different dietary protein sources related to type 2 diabetes (T2D) and determined whether the association is mediated by insulin resistance (IR) and β-cell dysfunction in a population-based cross sectional study of 4,427 women and 2,394 men aged 20–74 years in northeast China. We observed that the intake of total protein, animal protein, and red meat protein was positively associated with T2D prevalence in women. Comparing the women in the highest quintile of protein intake with those in the lowest quintile, the multivariable-adjusted odds ratios of T2D were 2.13 [95% confidence interval (CI): 1.18–3.81] for total protein, 2.27 (95% CI: 1.18–4.35) for animal protein, and 1.75 (95% CI: 1.14–2.68) for red meat protein. Mediation analyses indicated that these associations were mediated mainly by the IR as measured by the homeostasis model (HOMA-IR). The proportions via the mediation of HOMA-IR were 29.0% (95% CI: 10.3%–55.5%), 35.0% (95% CI: 12.9%–83.3%), and 17.2% (95% CI: 5.2%–44.8%) for total protein-, animal protein-, and red meat protein–T2D associations, respectively. These findings support the notion that modifying the sources of dietary protein may be potentially applied to prevent T2D.

Type 2 diabetes (T2D) is the consequence of a complex interplay of genetic and environmental factors^1^. Dietary modification is thought to be as a most effective strategy to prevent and treat T2D^2^,^3^. However, the role of specific dietary composition of macronutrients in relation to T2D remains uncertain. Although a large body of observational studies have examined dietary fat and carbohydrate affecting T2D risk^4^, relatively few studies have directly investigated the role of dietary protein in relation to T2D risk. Short–term intervention studies have revealed that protein intake positively affects body weight control and promotes insulin secretion in pancreatic β-cells^5^,^6^,^7^,^8^. In contrast, large and long-term observational studies generally pointed in the opposite direction relating the effect of protein intake to insulin sensitivity and T2D risk^9^,^10^,^11^,^12^. The possible underlying mechanisms involved in relating protein intake to T2D risk also remain unclear. In one short-term intervention study involving 15 men, dietary protein intake elicits an insulinotropic effect promoting insulin secretion^6^. Conversely, several animal studies have reported that dietary protein induces insulin resistance (IR) in peripheral tissues^13^ and accelerates pancreas fatigue; as a consequence, T2D occurs^14^,^15^.

The prevalence of T2D increased substantially to epidemic proportion in China from 0.9% in 1980 to 11.6% in 2010. Rapid nutritional and lifestyle changes are likely responsible for this transitional pattern of diabetes in China^16^. According to the recent Chinese national nutrition survey, the proportion of energy from animal food rich in fat and protein has increased from 9.3% to 13.7% in 10 years^17^. However, no studies have yet directly and comprehensively investigated the amount of specific sources of dietary protein in relation to T2D risk in Chinese population. Therefore, we conducted a population-based study in Northeast China to examine the association of total and specific dietary protein intake with T2D prevalence directly. Specifically, we also adopted the strategy of mediation analysis to quantify the extent to which either IR or β-cell dysfunction may contribute to the protein–T2D relation.

## Results

### Characteristics of the participants

The representative sample of this cross-sectional study consisted of 6,821 participants (4,427 women and 2,394 men) whose characteristics are shown in Table 1 according to the energy-adjusted total protein intake quintiles by sex. The actual daily dietary intake is also described in Table 1, although only the energy-adjusted nutrients intake was used in the following statistical analyses. In our population, the protein from animal food sources accounted for 39% of the total protein intake. The proportions of the main animal protein sources were as follows: red meat (31%), egg (24%), seafood (17%), poultry (15%), and dairy (10%). The main plant protein sources were rice (42%), wheat (26%), vegetable (15%), and legumes (11%). The women with higher energy-adjusted total protein consumption were younger, less likely to be postmenopausal, characterized by higher serum levels of fasting glucose and HbA1c, homeostasis model assessment of IR (HOMA-IR), higher animal protein, fat, and cholesterol intake, and lower carbohydrate and plant protein intake. The men with higher energy-adjusted intake of total protein had higher serum 2 h glucose in the oral glucose tolerance test, fasting insulin and HbA1c levels, higher animal protein, fat, and cholesterol intake, and lower carbohydrate and plant protein intake.

### Associations between energy-adjusted protein intakes and T2D

As shown in Table 2, the prevalence of T2D increased significantly over the quintiles of energy-adjusted total protein intake in women. The prevalence rates of T2D were 9.4% and 4.3% in women with the highest and lowest intake of protein, respectively. Comparing the women in the highest with the lowest quintiles of intake, the age-adjusted odds ratio (OR) of T2D was 2.26 [95% confidence interval (CI): 1.30–3.94; *P* for trend < 0.01] for total protein intake. Even after adjustment for known T2D risk factors, including age, total energy intake, energy-adjusted intake of saturated fat, monounsaturated fat, polyunsaturated fat, cholesterol, fiber, physical activity, smoke, drink, family history of diabetes, economic status, education, body mass index (BMI), and waist, the positive association between total protein intake and T2D remained substantial [comparing the two extreme quintiles, multivariate-adjusted OR (95% CI): 2.13 (1.18–3.81), *P* for trend < 0.01]. Treated as a continuous variable, high total protein intake was associated with a 24% higher T2D prevalence for every 10- g increment in women [multivariable-adjusted OR (95% CI): 1.24 (1.03–1.41)]. The analyses of animal protein intake showed comparable results. When comparing the highest with the lowest animal protein intake in women, the multivariable-adjusted OR was 2.27 (95% CI, 1.18–4.35; *P* for trend = 0.02). However, in the analysis of plant protein and T2D, no significant association was observed in women regardless of the adjustment for T2D risk factors.

Although we observed that men with higher total protein and animal intakes but no plant protein intake also had relatively higher prevalence of T2D (10.5% versus 8.4% and 10.7% versus 8.8% for the two extreme quintiles of total protein and animal protein intakes, respectively), the adjusted-ORs were not statistically significant.

When different sources of animal protein were further examined in relation to T2D prevalence, only protein from red meats was significantly and positively associated with T2D in women [comparing the two extreme quintiles, multivariable-adjusted OR (95% CI): 1.75 (1.14–2.68), *P* for trend = 0.002]. The association between 10- g increment of red meat protein intake and T2D was also confirmed [multivariate-adjusted OR (95% CI): 1.09 (1.01–1.17)] (Table 3).

Substituting 1% energy from total protein for equal amount of total carbohydrate was associated with elevated prevalence of T2D [multivariable-adjusted OR (95% CI): 1.06 (1.01–1.11)] (Table 4). For substitution of 1% energy from animal protein for plant protein, we observed a multivariable-adjusted OR of 1.05 (1.01–1.10) for T2D. Similar results were observed after substitution of 1% energy from red meat protein for protein from egg, seafood, and poultry. The substitution of red meat protein for dairy protein was not statistically significant.

### Mediation analysis

We investigated the mediator roles of IR (indicated by HOMA-IR) and β-cell dysfunction (indicated by HOMA-β) in the associations of total protein, animal protein, and red meat protein intakes with prevalence of T2D in women using mediation analysis. Results in Table 5 show that compared with the lowest total protein intake, the highest total protein intake was associated with 3.6% (95% CI: 1.8%–5.9%) increase in the prevalence of T2D after adjustment for T2D risk factors. Of this total effect, 29.0% (95% CI: 10.3%–55.5%) was mediated by HOMA-IR. Similarly, with the lowest animal protein intake as reference, the highest intake of animal protein and red meat protein accounted for 3.7% and 4.4% increase, respectively, in T2D prevalence. Of these total effects, 35.0% and 17.2% were mediated by HOMA-IR, respectively (Table 5).

The estimates in the above-mentioned mediation analysis were identified when the sequential ignorability assumption holds. However, since excluding the existence of unobserved variables that confound the relation of mediator and outcome seems impossible, it is unlikely this assumption holds. We therefore conducted a sensitivity analysis assessing the product of coefficients of determination (R^2^) from the mediation analysis to address what would happen to the estimates when unobserved confounders existed. Findings from sensitivity analysis indicated that when the unobserved confounders explained 20% of residual variance (R^2*^) or 10% of total variance (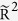) in the mediator and outcome then the mediation effect would be zero (Table 5).

## Discussion

In this study, we confirmed that higher intakes of total protein and animal protein, especially red meat protein, were associated with higher prevalence of T2D in Chinese women, but not in men. Substitutions of total protein for total carbohydrate, animal protein for plant protein, and protein from red meat for protein from egg, seafood, and poultry were associated with the increased prevalence of T2D. These findings demonstrated that the association between dietary protein intake and prevalence of T2D depended on the food sources of protein and sex difference. Furthermore, we discovered that IR, and not β-cell dysfunction, mediated the above-mentioned associations between T2D and total protein, animal protein, and red meat protein.

Our study supports those of previous studies, which reported the association between total and animal protein intake and T2D in American and European populations^11^,^18^,^19^. Recently, results from the Nurses’ Health Study (NHS), NHS II, and Health Professionals Follow-up Study (HPFS) showed that higher intake of total and animal protein was associated with the 7% and 13% increased risk of T2D, respectively, comparing the extreme quintiles of protein intake^19^. The Women’s Health Initiative found that the biomarker-calibrated protein intake was positively associated with diabetes risk^18^. Similar associations were also reported in the European cohort of EPIC-Interact case-cohort study^11^. In these studies, higher dietary protein intake was also related with higher BMI, and the association between protein intake and T2D risk was attenuated after adjustment for BMI, suggesting that body weight may partly mediate the association between total and animal protein and T2D risk. However, in the current study, we found that total and animal protein intake was positively associated with the prevalence of T2D at the absence of elevated body weight or waist. Though it is unclear why body weight and waist was not associated with protein intake in this study, the results suggested that, at least, the association between total and animal protein and T2D should not be simply attributed to the body weight changes. In addition, the associations between total and animal protein and T2D were reported to be stronger in non-obese participants than in obese participants^9^,^19^, also implying that such associations cannot be just explained by body weight gain.

In this study, we reported that total protein was positively associated with the prevalence of T2D, although the overall protein intake is relatively low compared with that of many western countries and cannot explain fully the high prevalence of T2D in China. The high prevalence of T2D in China is thought to be caused by many risk factors, including urbanization, obesity, physical inactivity, diet, early-life exposure to malnutrition, and genetic factors^20^. Nevertheless, protein consumption is just one of the dietary factors. Thus, the contribution of protein consumption on the high prevalence of T2D in China is very limited.

Until now, the findings regarding the association between plant protein and T2D are not consistent. A recent pooled analysis of NHS, NHS II, and HPFS showed a modest inverse association between dietary plant protein intake and risk of T2D; however, this association was nonsignificant in the separate analyses of these three cohorts^19^. Additionally, both the EPIC-Interact case-cohort study and EPIC-NL study also did not report significant associations between plant protein intake and risk of T2D^9^,^11^. In our study, the association between intake of plant protein and T2D also did not reach the significant level. The discrepancy among these reported plant protein–T2D associations may be attributed to the relatively modest association between plant protein intake and T2D, which only was observed in the large-scale pooled analysis (4,146,216 person-years of follow-up among 205,802 participants)^19^.

In view of the association of animal protein intake with T2D in our study, we further determined the relations of T2D with the protein intake from main animal protein sources, including red meat, egg, seafood, poultry, and dairy. Our results, which are consistent with previous studies^21^,^22^,^23^, indicated that only the protein from red meat was significantly associated with T2D. Furthermore, we found that the substitution of plant protein for animal protein or substitutions of red meat protein for other animal proteins were associated with increased prevalence of T2D. Therefore, even though the total dietary protein intake remains unchanged, modifying the protein sources, for example, substituting plant protein for animal protein or substituting dietary protein from egg, seafood, or poultry for red meat protein, is supposed to be helpful for controlling the prevalence of T2D.

The sex-specific difference observed for the associations between protein intake and T2D needs to be interpreted with caution, although it has been consistently reported in prior studies^9^,^11^. Although why animal protein intake was found to be positively associated with T2D in women but not in men is not entirely clear, differences in dietary and lifestyle factors between men and women as well as sex differences in T2D mechanisms, which may account for the sex difference in protein–T2D relation, have been well-documented^24^,^25^. In our study, the energy-adjusted protein intake was consistent in men and women, and no statistically significant differences were observed in the distribution of dietary and lifestyle factors between men and women. A global diabetes survey showed that the sex difference in the prevalence of diabetes was reversed depending on the stage of productive life^26^, indicating that men are predisposed to diabetes before puberty, whereas more women are diabetic after menopause when serum estrogen levels decrease^27^. In our study, half of the women are postmenopausal and in the stage of T2D susceptibility, which might have aggravated the effect of animal protein intake on T2D and then contributed to the sex differences in the relation of protein–T2D. In addition, in our study, given that the sample size of women was almost twice that of men, it may be used to explain the lack of statistical significance of findings in men. However, our power analysis showed that the expected effect sizes should be similar between men and women.

On the basis of clarifying the association between dietary protein intake and T2D in Chinese population, further examining the potential mechanisms involved in this association is valuable. Given that IR and β-cell dysfunction are the two main features in the pathogenesis of T2D^28^,^29^,^30^, speculating that the association between dietary protein intake and T2D should be partly mediated by IR and/or β-cell dysfunction is reasonable. Our mediation analysis attempted to quantify the extent to which IR and/or β-cell dysfunction contribute to the association between dietary protein intake and T2D. Results showed that the observed positive associations between intakes of total, animal, and red meat protein and T2D in women were mainly mediated by IR but not β-cell dysfunction.

Previous findings about short-term and long-term effects of high dietary protein intake on insulin action are inconclusive. Generally, in short-term studies, dietary proteins have an insulinotropic effect and thus promote insulin secretion, which improves IR^6^,^31^. However, a long-term study showed that consumption of a high-protein diet for six months decreased insulin sensitivity in healthy subjects^32^. Given the IR, wherein the tissues are no longer sensitive to the physiological actions of insulin and which occurs when tissues are chronically overexposed to high levels of insulin^33^, speculating that consumption of a high-protein diet may lead to hyperinsulinemia and in the long term may cause IR is plausible.

As to the physiological mechanism underlying the relation of protein and T2D, current studies mainly focus on branched-chain amino acids (BCAA) inducing IR via the mechanistic target of rapamycin (mTOR) pathway. Recent human studies demonstrated that plasma BCAA level^34^,^35^ and dietary consumption of BCAA^36^ were positively related to incidence of IR and/or T2D^37^,^38^. Furthermore, a high-fat BCAA diet led to IR in rats through activation of the mTOR pathway^39^, which is a nutrient-sensing pathway and integrates signals from nutrients, such as amino acids and glucose, especially BCAA, and insulin signaling to regulate cell growth and metabolism^40^. The activation of mTOR by nutrients can cause phosphorylation of the insulin receptor substrate 1 (IRS1). Suppression of IRS1 function by mTOR activation may lead to decreased insulin sensitivity. However, direct evidence supporting the causal relationship among dietary protein intake, circulating BCAA, and IR is needed in further research.

These findings should be considered in the light of their limitations. First, this study is cross-sectional only. Therefore, notably, establishing causal relationship is impossible, and even reverse causality may exist. A large-scale longitudinal study and long-term intervention study are warranted to confirm the causal relationship between dietary protein intake and T2D. Second, although we adjusted a number of potential confounders in analyses, the possibility of residual confounding cannot be dismissed. Therefore, the observed protein-T2D relation in this study should be explained while considering the potential residual confounding. Third, results from the mediation analysis conducted in the cross-sectional study cannot be interpreted as causal and just represent association. Thus, HOMA-IR cannot be interpreted as an actual pathway for the assumed effect of dietary proteins.

In conclusion, higher intakes of total, animal, and red meat protein were associated with higher prevalence of T2D in Chinese women but not in men. Moreover, these associations were mediated by IR but not by β-cell dysfunction. These data from a large population-based study support the notion that different dietary protein sources may play an important role in affecting T2D risk, particularly in women, and that further work should examine the balance of benefits and risks in modifying protein sources for the prevention of T2D in Chinese women and men.

## Subjects and Methods

### Study population

The data were obtained from a population-based cross-sectional T2D survey, which was conducted in Harbin, China. The detailed study design had been reported elsewhere^41^. Briefly, a total of 8,940 individuals aged 20–74 years were recruited using a stratified multistage random cluster sampling design. In this study, the inclusion criteria were as follows: 1) provided written informed consent, 2) without prior diagnosis of diabetes, 3) without unusual total energy intake (i.e., daily energy intake > = 800 kcal/d and = < 4,500 kcal/d for men or > = 500 kcal/d and = < 4,000 kcal/d for women), and 4) absence of liver diseases, thyroid dysfunction, hematological diseases, or chronic kidney disease. Finally, a total of 6821 individuals, including 2394 men and 4427 women, met the inclusion criteria. Significant differences were not observed in the basic characteristics between the included and excluded participants (Supplementary Table 1). This study was approved by the Ethics Committee of Harbin Medical University and in accordance with the Declaration of Helsinki. The informed consent was obtained from all subjects.

### Assessment of protein intake

We used a validated food-frequency questionnaire (FFQ), including 103 food items from 14 food groups, to assess the dietary intake of the study participants for over one year before enrollment. Individual daily intake of protein and other nutrients were calculated using the Food Nutrition Calculator V1.60 (Chinese Center for Disease Control, Beijing, China). Intake of each nutrient was adjusted for total energy intake by using the regression residual method^42^. Total protein intake was considered as the sum of animal protein and plant protein intakes. Animal protein included the protein from red meat (pork, beef, and lamb), poultry (chicken, duck, and goose), dairy (cow’s milk, yogurt, and milk powder), eggs, and seafood (carp, crucian, hairtail, yellow croaker, and shrimp). Plant protein included the protein from rice, wheaten food, potatoes and its products, legumes and its products, vegetables, and fruits. The validation and detailed food items of the FFQ have been reported elsewhere^41^,^43^. The energy-adjusted correlation coefficient between FFQ and a three-day dietary record was 0.51–0.69 for the macronutrients, including protein^43^.

### Anthropometric measurement and biochemical assessment

Individual demographics were collected using a structured questionnaire about age, sex, educational level, cigarette smoking, alcohol use, physical activity, economic status, previous medical history, and family history. All anthropometric measurements, including height, fasting body weight, and waist circumferences, were taken by trained interviewers according to the standard procedures^41^,^43^. The above measurements were performed twice, and their means were used for analysis. BMI was calculated using the equation: BMI = weight (kg)/height (m^2^).

Participants without self-reported diabetes underwent a 75- g oral glucose tolerance test. Both fasting and 2- h serum glucose were measured using an automatic biochemistry analyzer (Hitachi 7100, Tokyo, Japan). Fasting serum insulin was detected by using a ROCHE Elecsys 2010 Chemiluminescence Immune Analyzer (Roche Diagnostics). All samples were analyzed by an experienced laboratory technician, who was blinded to the study design. IR index (HOMA-IR) and basal β-cell function index (HOMA-β) were calculated using the following equations:


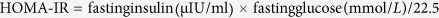



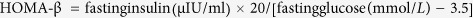


According to the 1999 World Health Organization diagnostic criteria, T2D was defined as fasting glucose ≥7.0 mmol/L or 2 h glucose ≥11.1 mmol/L.

### Statistical analysis

Continuous variables with normal distribution were given as means and standard deviation and with skewed distribution, were provided as medians and interquartile ranges. HOMA-IR and HOMA-β were normalized by a natural logarithm transformation before analysis. Categorical variables were shown in proportions.

Protein intake, adjusted for total energy intake by the regression residual method^42^, was categorized into quintiles. Logistic regression models were used to calculate ORs and 95% CI for the associations between T2D and quintiles of energy-adjusted protein intake. We estimated *P* for trend by including the median of different energy-adjusted protein intake per quintile as continuous variables in the logistic regression models. We also analyzed the association between energy-adjusted protein intake per 10-g increment and T2D. In multivariate models, we first adjusted for age. In the second model, we added the nutritional factors: energy-adjusted intake of saturated fat, monounsaturated fat, polyunsaturated fat, fiber, cholesterol, and total energy. In the third model, we further adjusted for the T2D risk factors, including physical activity (inactive, moderately active, or active), smoking status (never, former, or current), alcohol consumption (yes or no), education (low, secondary, or high), economic status (low or high), and family history of diabetes (yes or no). In the fourth model, BMI and waist measurements were included.

We performed power analyses for the primary exposure-outcome relation, i.e., the association between total protein intake and the prevalence of T2D. In these analyses, for women, we assumed a prevalence rate of 0.05 for T2D in the lowest quintile of total protein intake, and the number of subjects in each quintile was 900. This study had 80% power to detect an OR of 1.7. For men, we assumed a prevalence rate of 0.1 for T2D in the lowest quintile of total protein intake, and the number of subjects in each quintile was 500. The power for detecting an OR of 1.7 was 80%.

We also estimated the association between substituting total protein for an equal exchange of carbohydrate and T2D and simulated the isocaloric substitution of animal protein for plant protein and isocaloric substitution of red meat protein for proteins from other animal food sources, such as egg, dairy, seafood, and poultry. To fit these models, we simultaneously included total energy, percentage of energy derived from total protein, animal protein or red meat protein, and the substitution nutrients of interest as continuous variables along with the covariates listed in Model 4. The differences between the coefficients for the two nutrients of interest were used to estimate the OR for the association of substituting 1% of energy intake. The 95% CIs were derived from the variances and covariance matrix of the coefficients^19^.

To investigate whether IR or β-cell dysfunction mediated the association between dietary protein intake and T2D, we conducted mediation analyses by considering protein intake as a predictor and by analyzing HOMA-IR and HOMA-β as mediators. The total effects, direct effects, indirect effects, proportion via mediation, and their 95% CIs were obtained from nonparametric bootstrapping with 1000 iterations implemented through the *mediate* function in the *mediation* package in R^44^. In the mediation analysis, the confounding factors in the above-mentioned Model 3 were adjusted. The mediation analysis used in this study relies on the sequential ignorability assumption for point identification^44^,^45^. Given excluding the existence of unobserved variables that confound the relationship between mediator and outcome is virtually impossible, the sequential ignorability assumption is not satisfied in many studies. In this study, a sensitivity analysis was conducted to quantify the possible effect of the presence of unobserved confounders on the estimates. The influence of the unobserved confounders was expressed using the coefficients of determination of R^2*^ and 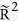, which represent the proportions of residual variance (R^2*^) and total variance (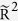) in the mediator and outcome explained by the hypothesized unobserved confounder^46^.

All statistical analyses were performed using R 3.2.2 (The R Foundation for Statistical Computing, Vienna, Austria). Two-sided *p* < 0.05 was considered to be statistically significant.

## Additional Information

**How to cite this article**: Li, J. *et al*. Dietary Protein Intake and Type 2 Diabetes Among Women and Men in Northeast China. *Sci. Rep.* **6**, 37604; doi: 10.1038/srep37604 (2016).

**Publisher’s note:** Springer Nature remains neutral with regard to jurisdictional claims in published maps and institutional affiliations.

## Supplementary Material

Supplementary Information

## Figures and Tables

**Table 1:** Sex-specific characteristics and dietary consumption by categories of energy-adjusted total protein intake (*n* = 6,821).

	Women (n = 4, 427)			Men (n = 2, 394)		
	Q1: 57.1 g/d (52.5, 57.1)^a^	Q3: 70.4 g/d (69.3, 71.5)^a^	Q5: 86.6 g/d (82.1, 95.6)^a^	Q1: 57.1 g/d (52.0, 60.5)^a^	Q3: 72.0 g/d (70.7, 73.4)^a^	Q5: 90.9 g/d (85.9, 101.4)^a^
N	885	885	886	479	479	478
Age (yr)	50.2 ± 10.2	49.3 ± 9.9	48.6 ± 10.3	49.3 ± 11.4	49.8 ± 10.5	48.2 ± 11.2
BMI (kg/m^2^)	24.6 ± 3.5	24.3 ± 3.4	24.5 ± 3.5	25.7 ± 3.2	25.6 ± 3.3	25.7 ± 3.6
Waist (cm)	82.5 ± 9.4	82.2 ± 9.1	82.2 ± 9.2	90.8 ± 9.2	90.7 ± 9.7	91.8 ± 10.0
Fasting glucose (mmol/L)	4.6 ± 1.1	4.7 ± 1.1	4.7 ± 1.2	4.8 ± 1.3	4.9 ± 1.4	4.9 ± 1.4
2 h Glucose (mmol/L)	6.2 ± 2.4	6.3 ± 3.0	6.3 ± 2.8	6.3 ± 3.1	6.4 ± 2.8	6.7 ± 3.2
Fasting insulin (μU/ml)	8.0 ± 4.2	7.9 ± 4.3	8.4 ± 4.6	7.7 ± 4.8	7.7 ± 4.4	8.6 ± 5.0
HbA1c (%)	4.8 ± 0.6	4.8 ± 0.6	4.9 ± 0.7	4.8 ± 0.6	4.9 ± 0.6	5.0 ± 0.8
HOMA-IR	1.3 (0.8, 2.1)	1.4 (0.9, 2.1)	1.6 (1.0, 2.4)	1.4 (0.9, 2.1)	1.3 (0.9, 2.0)	1.4 (0.9, 2.2)
HOMA-β	134.1 (83.4, 244.4)	135.8 (80.7, 221.4)	131.5 (74.5, 226.9)	116.7 (60.6, 220.9)	112.3 (63.0, 196.6)	120.3 (63.8, 216.0)
Education level						
Higher than college (%)	31.6	32.4	37.6	36.3	42.4	45.2
Physical activity						
Active (%)	13.4	11.7	11.2	24.4	21.5	19.2
Smokers (%)	5.0	4.1	3.8	19.8	21.4	17.0
Drinking (%)	20.0	21.2	22.0	61.6	63.3	66.3
Economic status						
High economic status (%)	51.2	52.1	52.0	54.3	54.8	54.5
Family history of diabetes (%)	14.7	17.6	15.0	10.2	10.0	12.3
Postmenopausal (%)	62.3	58.2	51.7	/	/	/
Dietary Intake^b^						
Total energy (kcal/d)	2360.7 ± 653.3	1688.7 ± 567.9	2139.3 ± 711.8	2886.0 ± 751.3	1976.0 ± 670.2	2551.5 ± 843.4
Total protein (g/d)	63.7 ± 19.5	56.2 ± 19.1	93.9 ± 35.2	81.7 ± 22.7	67.7 ± 22.8	113.2 ± 40.1
Total protein (energy%)	10.7 ± 0.9	13.3 ± 0.4	17.7 ± 3.2	10.0 ± 0.9	13.7 ± 0.4	17.9 ± 2.9
Animal protein (g/d)	17.0 ± 10.6	21.0 ± 10.3	53.0 ± 31.8	21.4 ± 10.4	25.8 ± 12.1	65.2 ± 34
From red meat (g/d)	5.6 ± 6.0	6.7 ± 6.6	12.9 ± 11.3	8.0 ± 6.8	9.9 ± 8.8	18.6 ± 15.2
From poultry (g/d)	2.3 ± 3.1	3.2 ± 4.1	10.7 ± 13.7	3.2 ± 3.4	3.9 ± 3.8	12.9 ± 12.9
From dairy (g/d)	2.0 ± 2.7	2.6 ± 2.7	3.3 ± 3.2	1.7 ± 2.3	2.1 ± 2.6	2.7 ± 3.1
From egg (g/d)	3.9 ± 3.5	5.1 ± 3.4	9.1 ± 8.6	5.0 ± 4.1	5.5 ± 3.6	9.6 ± 7.5
From seafood (g/d)	2.2 ± 2.3	3.1 ± 2.8	15.9 ± 23.9	2.7 ± 2.6	3.9 ± 3.6	20.2 ± 29.3
Plant protein (g/d)	46.5 ± 15.2	35.1 ± 12.6	40.8 ± 16.1	60.0 ± 17.9	41.7 ± 14.6	47.8 ± 19.3
From rice (g/d)	25.0 ± 12.5	14.3 ± 8.6	12.0 ± 8.4	36.1 ± 14.7	17.4 ± 10.1	15.6 ± 11.5
From wheat (g/d)	9.2 ± 8.6	9.4 ± 6.9	10.4 ± 7.7	12.0 ± 10.6	12.6 ± 9.9	13.5 ± 10.6
From potato (g/d)	1.3 ± 1.2	0.6 ± 0.5	0.7 ± 0.7	1.2 ± 1.3	0.6 ± 0.5	0.8 ± 0.8
From legumes (g/d)	2.8 ± 3.3	3.3 ± 3.7	7.6 ± 7.5	3.5 ± 3.5	4.2 ± 3.8	9.1 ± 9.4
From vegetable (g/d)	5.4 ± 4.0	5.8 ± 4.2	8.2 ± 7.3	5.5 ± 4.1	5.8 ± 4.3	7.3 ± 6.5
From fruit (g/d)	2.0 ± 2.1	1.2 ± 1.0	1.4 ± 1.2	1.2 ± 1.4	0.9 ± 0.8	1.0 ± 0.9
Total fat (g/d)	28.8 ± 13.6	28.7 ± 13.6	53.4 ± 25.5	36.1 ± 13.9	35.3 ± 16.8	66.0 ± 28.7
Total fat (energy%)	10.8 ± 3.6	15 ± 3.9	22.3 ± 6.6	11.2 ± 3.0	15.8 ± 4.1	23.5 ± 6.5
Saturated fat (g/d)	7.3 ± 4.1	8.2 ± 4.3	15.6 ± 8.0	9.5 ± 4.3	10.3 ± 5.4	19.6 ± 9.2
Monounsaturated fat (g/d)	9.7 ± 5.5	10.9 ± 5.6	20.8 ± 11.0	12.7 ± 5.9	14.1 ± 7.2	26.6 ± 12.7
Polyunsaturated fat (g/d)	5.1 ± 1.9	4.9 ± 1.9	8.4 ± 3.5	6.8 ± 2.2	6.2 ± 2.3	10.5 ± 3.9
Total carbohydrate (g/d)	461.6 ± 126.3	301.5 ± 99.3	320.8 ± 113.6	558.7 ± 146.5	346.9 ± 115.2	376.1 ± 137.2
Total carbohydrate (energy%)	78.4 ± 4.1	71.7 ± 4.0	60.0 ± 8.1	77.5 ± 3.4	70.5 ± 4.2	58.6 ± 7.5
Cholesterol (mg/d)	231.4 ± 163.9	295.6 ± 157.7	608.5 ± 407.8	295.3 ± 187.5	334.9 ± 169	686.4 ± 359.4
Fiber (g/d)	15.6 ± 6.7	12.2 ± 5.6	15.1 ± 8.3	12.0 ± 4.8	13.0 ± 4.2	12.4 ± 6.9

Data were presented as mean ± standard deviation, median (25th percentile, 75th percentile), or proportions.

^a^FFQ-estimated intake, adjusted for total energy by the residual method.

^b^Actual daily nutrients intake estimated with FFQ without adjustment for total energy.

**Table 2:** The odds ratios of type 2 diabetes according to quintiles of energy-adjusted protein intake by sex.

	Protein Quintiles, OR (95% CI)					*P* for trend	Per 10 g
	Q1 (low)	Q2	Q3	Q4	Q5 (high)		
Women							
Total protein *N* (cases)	885 (38)	886 (46)	885 (57)	885 (64)	886 (83)		
Model 1	1	1.35 (0.58, 1.40)	1.56 (1.01, 2.45)	1.73 (1.07, 1.82)	2.26 (1.30, 3.94)	<0.001	1.32 (1.14, 1.49)
Model 2	1	1.19 (0.52, 1.34)	1.51 (0.95, 2.40)	1.70 (1.04, 2.79)	2.20 (1.24, 3.89)	<0.001	1.28 (1.07, 1.45)
Model 3	1	1.19 (0.51, 1.33)	1.49 (0.92, 2.39)	1.68 (1.02, 2.77)	2.15 (1.21, 3.83)	<0.001	1.27 (1.05, 1.43)
Model 4	1	1.18 (0.50, 1.32)	1.48 (0.91, 2.37)	1.66 (1.00, 2.72)	2.13 (1.18, 3.81)	<0.001	1.24 (1.03, 1.41)
Animal protein *N* (cases)	885 (33)	886 (53)	885 (56)	886 (63)	885 (83)		
Model 1	1	1.65 (1.04, 2.67)	1.73 (1.58, 2.55)	1.93 (1.17, 3.23)	2.54 (1.40, 4.68)	<0.001	1.41 (1.30, 1.49)
Model 2	1	1.58 (0.97, 2.58)	1.68 (1.08, 2.61)	1.85 (1.09, 3.15)	2.39 (1.26, 4.52)	0.02	1.36 (1.26, 1.45)
Model 3	1	1.57 (0.96, 2.57)	1.68 (1.08, 2.60)	1.84 (1.08, 3.16)	2.36 (1.25, 4.52)	0.02	1.34 (1.24, 1.45)
Model 4	1	1.50 (0.91, 2.48)	1.61 (1.03, 2.51)	1.80 (1.05, 3.10)	2.27 (1.18, 4.35)	0.02	1.33 (1.23, 1.42)
Plant protein *N* (cases)	885 (50)	886 (42)	885 (65)	885 (74)	886 (57)		
Model 1	1	0.85 (0.57, 1.30)	1.32 (0.85, 2.07)	1.52 (0.94, 2.45)	1.17 (0.69, 2.04)	0.47	1.08 (0.87, 1.37)
Model 2	1	0.76 (0.47, 1.21)	1.27 (0.80, 2.02)	1.49 (0.91, 2.42)	1.09 (0.61, 1.96)	0.31	0.97 (0.75, 1.26)
Model 3	1	0.76 (0.47, 1.22)	1.26 (0.80, 2.00)	1.47 (0.90, 2.40)	1.08 (0.60, 1.95)	0.33	0.96 (0.74, 1.26)
Model 4	1	0.74 (0.46, 1.20)	1.28 (0.80, 2.04)	1.47 (0.89, 2.40)	1.08 (0.59, 1.95)	0.35	0.96 (0.74, 1.25)
Men							
Total protein *N* (cases)	479 (40)	479 (49)	479 (36)	479 (46)	478 (50)		
Model 1	1	1.27 (0.82, 1.98)	0.89 (0.56, 1.43)	1.16 (0.74, 1.82)	1.31 (0.85, 2.04)	0.30	1.06 (0.99, 1.14)
Model 2	1	1.35 (0.83, 2.20)	0.95 (0.55, 1.65)	1.22 (0.69, 2.16)	1.20 (0.63, 2.30)	0.73	1.03 (0.94, 1.15)
Model 3	1	1.34 (0.82, 2.20)	0.95 (0.54, 1.65)	1.22 (0.69, 2.15)	1.18 (0.61, 2.28)	0.75	1.03 (0.93, 1.14)
Model 4	1	1.35 (0.83, 2.21)	0.94 (0.53, 1.64)	1.22 (0.70, 2.17)	1.17 (0.60, 2.27)	0.80	1.04 (0.93, 1.15)
Animal protein *N* (cases)	480 (42)	478 (42)	479 (50)	479 (36)	478 (51)		
Model 1	1	1.04 (0.66, 1.63)	1.24 (0.81, 1.92)	0.89 (0.55, 1.41)	1.36 (0.88, 2.10)	0.25	1.06 (1.00, 1.13)
Model 2	1	1.02 (0.62, 1.70)	1.21 (0.70, 2.08)	0.81 (0.43, 1.51)	1.01 (0.48, 2.15)	0.88	1.04 (0.95, 1.14)
Model 3	1	1.03 (0.62, 1.70)	1.20 (0.69, 2.08)	0.80 (0.43, 1.50)	1.01 (0.48, 2.14)	0.84	1.04 (0.93, 1.14)
Model 4	1	1.04 (0.62, 1.72)	1.19 (0.68, 2.07)	0.81 (0.43, 1.52)	1.02 (0.47, 2.18)	0.85	1.05 (0.94, 1.15)
Plant protein *N* (cases)	479 (49)	479 (41)	479 (49)	479 (42)	478 (40)		
Model 1	1	0.82 (0.53, 1.28)	0.95 (0.62, 1.45)	0.74 (0.48, 1.16)	0.70 (0.45, 1.09)	0.11	0.88 (0.76, 1.02)
Model 2	1	0.94 (0.57, 1.54)	1.10 (0.66, 1.86)	0.87 (0.49, 1.55)	0.82 (0.42, 1.60)	0.55	0.93 (0.72, 1.19)
Model 3	1	0.93 (0.56, 1.52)	1.10 (0.66, 1.85)	0.86 (0.49, 1.54)	0.81 (0.42, 1.58)	0.56	0.93 (0.73, 1.19)
Model 4	1	0.91 (0.54, 1.49)	1.05 (0.62, 1.77)	0.84 (0.46, 1.49)	0.78 (0.39, 1.53)	0.49	0.91 (0.71, 1.17)

Model 1: adjusted age (continuous); Model 2: Model 1 + total energy intake, energy-adjusted intake (quintiles) of saturated fat, monounsaturated fat, polyunsaturated fat, cholesterol, and fiber; Model 3: Model 2 + physical activity (inactive, moderately active, or active), smoke (never, former, or current), drink (yes or no), family history of diabetes (yes or no), economic status (high or low), and education (low, secondary or high); Model4: Model3 + BMI (continuous) + waist (continuous).

**Table 3:** The odds ratios of type 2 diabetes according to quintiles of energy-adjusted animal protein intake with different sources in women^a^.

	Energy-adjusted animal protein quintiles, OR (95% CI)					*P* for trend	Per 10 g
	Q1 (low)	Q2	Q3	Q4	Q5 (high)		
Red meat protein	1	1.05 (0.67, 1.63)	1.57 (1.00, 2.47)	1.66 (1.04, 2.65)	1.75 (1.14, 2.68)	0.002	1.09 (1.01, 1.17)
Egg protein	1	1.30 (0.87, 1.96)	1.31 (0.89, 1.94)	1.10 (0.73, 1.67)	1.30 (0.87, 1.97)	0.41	1.12 (0.89, 1.39)
Seafood protein	1	0.96 (0.58, 1.78)	1.23 (0.77, 1.98)	0.95 (0.75, 1.84)	1.13 (0.72, 1.75)	0.57	0.78 (0.60, 1.01)
Poultry protein	1	0.97 (0.64, 1.48)	1.35 (0.86, 2.13)	1.05 (0.71, 1.59)	0.94 (0.59, 1.5)	0.98	0.99 (0.82, 1.19)
Dairy protein	1	0.83 (0.56, 1.23)	0.75 (0.42, 1.19)	1.14 (0.52, 1.86)	1.18 (0.81, 1.71)	0.12	1.13 (0.63, 1.86)

^a^The multivariate model was simultaneously adjusted for age (continuous), total energy intake, energy-adjusted intake (quintiles) of saturated fat, monounsaturated fat, polyunsaturated fat, cholesterol, fiber, physical activity (inactive, moderately active, or active), smoke (never, former, or current), drink (yes or no), family history of diabetes (yes or no), economic status (high or low), education (low, secondary or high), BMI (continuous), and waist (continuous).

**Table 4:** Multivariable-adjusted ORs (95% CI) for the association between protein intake and type 2 diabetes after substitution of 1% of energy from total protein for equal exchange of carbohydrate, substitution of animal protein for plant protein, and substitution of red meat protein for other animal proteins.

Substitution	OR (95% CI)^a^
Substitution total protein for carbohydrate	1.06 (1.01, 1.11)
Substitution animal protein for plant protein	1.05 (1.01, 1.10)
Substitution red meat protein for egg protein	1.04 (1.00, 1.09)
Substitution red meat protein for dairy protein	1.03 (0.99, 1.08)
Substitution red meat protein for seafood protein	1.06 (1.02, 1.11)
Substitution red meat protein for poultry protein	1.04 (1.00, 1.10)

^a^Adjusted for age (continuous), total energy intake, energy-adjusted intake (quintiles) of saturated fat, monounsaturated fat, polyunsaturated fat, cholesterol, fiber, physical activity (inactive, moderately active, or active), smoke (never, former, or current), drink (yes or no), family history of diabetes (yes or no), economic status (high or low), education (low, secondary or high), BMI (continuous), and waist (continuous).

**Table 5:** Association of energy-adjusted protein intake and type 2 diabetes with mediation of insulin resistance and insulin secretion in women^a^.

Mediators	Total effect, estimate, % (95% CI)	Indirect effect, estimate, % (95% CI)	Direct effect, estimate, % (95% CI)	Proportion via mediation, estimate, % (95% CI)	Sensitivity analysis	
					R^2*^	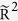
Association of energy-adjusted total protein intake and diabetes						
HOMA-IR	3.6 (1.8, 5.9)	1.0 (0.4, 1.7)	2.6 (0.9, 5.0)	29.0 (10.3, 55.5)	0.2	0.1
HOMA-β	3.8 (1.0, 6.6)	0.1 (−0.6, 0.8)	3.7 (1.1, 6.4)	2.4 (−27.9, 30.2)	0.2	0.1
Association of energy-adjusted animal protein intake and diabetes						
HOMA-IR	3.7 (0.4, 5.8)	1.1 (0.5, 1.8)	2.1 (0.6, 4.4)	35.0 (12.9, 83.3)	0.2	0.1
HOMA-β	3.2 (0.4, 6.0)	0.2 (−0.5, 0.9)	3.0 (0.2, 5.8)	8.8 (−35.6, 66.1)	0.2	0.1
Association of energy-adjusted intake of animal protein from red meat and diabetes						
HOMA-IR	4.4 (1.8, 6.8)	0.8 (0.2, 1.4)	3.6 (1.0, 6.2)	17.2 (5.2, 44.8)	0.2	0.1
HOMA-β	4.6 (1.8, 6.5)	0.5 (−0.03, 1.0)	3.4 (0.8, 6.4)	15.8 (−0.4, 51.2)	0.2	0.1

R^2*^, the proportion of residual variances and 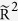, the proportion of original variances that were explained by the omitted confounding.

^a^The mediation analysis models were adjusted for age (continuous), total energy intake, energy-adjusted intake (quintiles) of saturated fat, monounsaturated fat, polyunsaturated fat, cholesterol, fiber, physical activity (inactive, moderately active, or active), smoke (never, former, or current), drink (yes or no), family history of diabetes (yes or no), economic status (high or low), and education (low, secondary or high).
