# Supplementary material for: Dietary Protein Intake and Type 2 Diabetes Among Women and Men in Northeast China
Source: Sci Rep. 2016 Nov 29;6:37604. doi: 10.1038/srep37604 (PMC5126628; doi:10.1038/srep37604)
Supplement: Supplementary Information [file srep37604-s1.pdf]

Dietary Protein Intake and Type 2 Diabetes Among Women and Men in Northeast China

Jie Li, [1,2] Changhao Sun, [1] Simin Liu, [2] Ying Li[1*]

[1]National Key Discipline, Department of Nutrition and Food Hygiene, School of Public Health, Harbin Medical University, Harbin, China; [2]Departments of Epidemiology and Medicine, Center for Global Cardiometabolic Health, Brown University, Providence RI

[*] **Corresponding author**: Ying Li, PhD, Department of Nutrition and Food Hygiene, School of Public Health, Harbin Medical University, 157 Baojian Road, Harbin 150081, China. Email: liying_helen@163.com

Supplementary Table 1. Characteristics of the included and excluded participants

	Included	Excluded	*P* value
N	6,821	2,119	
Sex (male%)	35.1	33.8	0.22
Age	49.4 ± 10.4	49.2 ± 10.3	0.43
BMI (kg/m^2)	24.8 ± 3.5	25.0 ± 3.4	0.05
Waist (cm)	85.4 ± 10.2	85.8 ± 9.7	0.09
Fasting glucose (mmol/L)	4.8 ± 1.2	4.8 ± 1.1	0.89
2 h Glucose (mmol/L)	6.4 ± 3.0	6.4 ± 2.8	0.82
Total energy (kcal/d)	2350.4 ± 746.2	2327.5 ± 737.7	0.17
Protein (g/d)	71.8 ± 30.0	70.6 ± 29.1	0.07
Fat (g/d)	37.3 ± 21.1	36.8 ± 20.7	0.24
Carbohydrate (g/d)	364.9 ± 138.1	361.9 ± 136.5	0.35
Smoker (%)	17.0	16.0	0.46
Drinking (%)	35.4	34.8	0.57
Active physical activity (%)	15.0	14.2	0.20

Data were presented as mean ± standard deviation or proportions. T test for continuous variables and chi-square test for categorical variables were applied to compare the differences between included and excluded participants.
